# Top Food Contributors of Sodium Intake in US Children and Adults Aged 1 Year or Older, National Health and Nutrition Examination Survey, August 2021–August 2023

**DOI:** 10.5888/pcd23.260054

**Published:** 2026-08-13

**Authors:** Alain K. Koyama, Rebecca C. Woodruff, Sandra L. Jackson, Janelle P. Gunn, Julie L. Self

**Affiliations:** 1 Division of Nutrition, Physical Activity and Obesity, Centers for Disease Control and Prevention, Atlanta, Georgia; 2 Division for Heart Disease and Stroke Prevention, Centers for Disease Control and Prevention, Atlanta, Georgia

## Abstract

To identify the food categories contributing most to sodium intake in the US, we used 2021–2023 National Health and Nutrition Examination Survey data. Approximately one-third of total dietary sodium intake among children and adults came from deli meat sandwiches (5.0%), pizza (4.4%), savory snacks (4.4%), soups (4.4%), poultry (3.8%), pasta mixed dishes (3.8%), bread (3.1%), and cheese (2.7%). Among subgroups, the highest contributor to sodium intake among children aged 1 to 11 years was savory snacks (approximately 8%) and among non-Hispanic Asian participants, soups (8.7%). Information on current top contributors to sodium intake can be leveraged to inform sodium reduction interventions.

**Summary**

**What is already known on this topic?**

Dietary sodium intake exceeds recommended daily levels for most children and adults in the US.

**What is added by this report?**

Overall, the 8 largest contributors to sodium intake were deli meat sandwiches, pizza, savory snacks, soups, poultry, pasta mixed dishes, bread, and cheese. Results were mostly consistent across subgroups of sex, hypertension status, and overweight/obesity, with differences among young children and some racial and ethnic groups.

**What are the implications for public health practice?**

Consumers, food manufacturers, policymakers, and others can focus on the largest contributors of sodium intake to inform interventions aimed at reducing excess sodium intake and the consequent risk of adverse health outcomes.

## Introduction

Excess sodium intake throughout the lifespan is associated with adverse health outcomes, including hypertension and cardiovascular disease later in life (1). Among children and adults combined (aged ≥1 y), 89% exceed recommended daily maximum intake levels of sodium (aged 1–3 y = 1,200 mg/day; 4–8 y = 1,500 mg/day; 9–13 y = 1,800 mg/day; ≥14 y = 2,300 mg/day) (2). Effective systems-level and behavioral interventions could promote reductions in daily sodium intake. Identifying the top food contributors to sodium intake can help inform these interventions.

The most recently published studies using data from the 2015–2016 (3) and 2017–2018 (4) National Health and Nutrition Examination Survey (NHANES) reported several food categories as the consistent top contributors to sodium intake overall among both children and adults, including bread, cold cuts, pizza, poultry, soups, and savory snacks. Our study aimed to update previous evidence to identify the food categories contributing most to sodium intake among US children and adults, overall and by demographic subgroups. Updated food categories in the most recent NHANES data allow more detailed categorization for several key food groups (eg, sandwiches, soups).

## Methods

### Study sample

We used data from the August 2021–August 2023 NHANES, a nationally representative survey of the noninstitutionalized US civilian population conducted by the Centers for Disease Control and Prevention’s National Center for Health Statistics (5). Interviewers obtained written informed consent from adults or consent from a parent or guardian for participants younger than 18 years. This analysis was conducted in accordance with applicable federal law and was not subject to institutional review board approval because data used were deidentified.

### Statistical analysis

Demographic variables were age (1–4 y, 5–11 y, 12–17 y, 18–39 y, 40–59 y, ≥60 y), sex (female, male), race and ethnicity (Hispanic, non-Hispanic Asian, non-Hispanic Black, non-Hispanic White, non-Hispanic Other [American Indian/Alaska Native, Native Hawaiian/Other Pacific Islander, and “other”]). Overweight or obesity was based on body mass index (≥85th percentile for those aged 2–19 y and ≥25 kg/m^2^ for those aged ≥20 y). Hypertension (self-reported use of hypertension medication, measured systolic pressure ≥130 mm Hg, or measured diastolic pressure ≥80 mm Hg) was measured for participants aged 18 years or older.

To estimate sodium intake, we used the first of two 24-hour dietary recalls recorded using the US Department of Agriculture’s (USDA’s) automated multiple-pass method (6). We categorized foods into 90 mutually exclusive categories adapted from USDA’s What We Eat in America (2021–2023) (7). The proportion of each food category contributing to daily sodium intake was defined as the sum of the sodium consumed from a given food category for all participants in the designated group divided by the sum of the sodium consumed from all food categories for all participants in the same group. Sodium included salt added during food preparation but not salt added at the table. Sodium density was calculated as milligrams of sodium per 1,000 kcal. We used R version 4.5.0 (R Foundation) for analyses, accounting for the complex survey design and estimating variance using Taylor series linearization. Survey weights from day 1 dietary recall were used.

## Results

Among 8,860 participants, 2,227 were excluded: 133 (1.5%) were younger than 1 year; 2,113 (23.8%) did not have a valid dietary recall or reported zero calories of intake; and 60 (0.7%) reported consuming human milk, resulting in an analytic sample of 6,633 participants.

**Figure 1:**
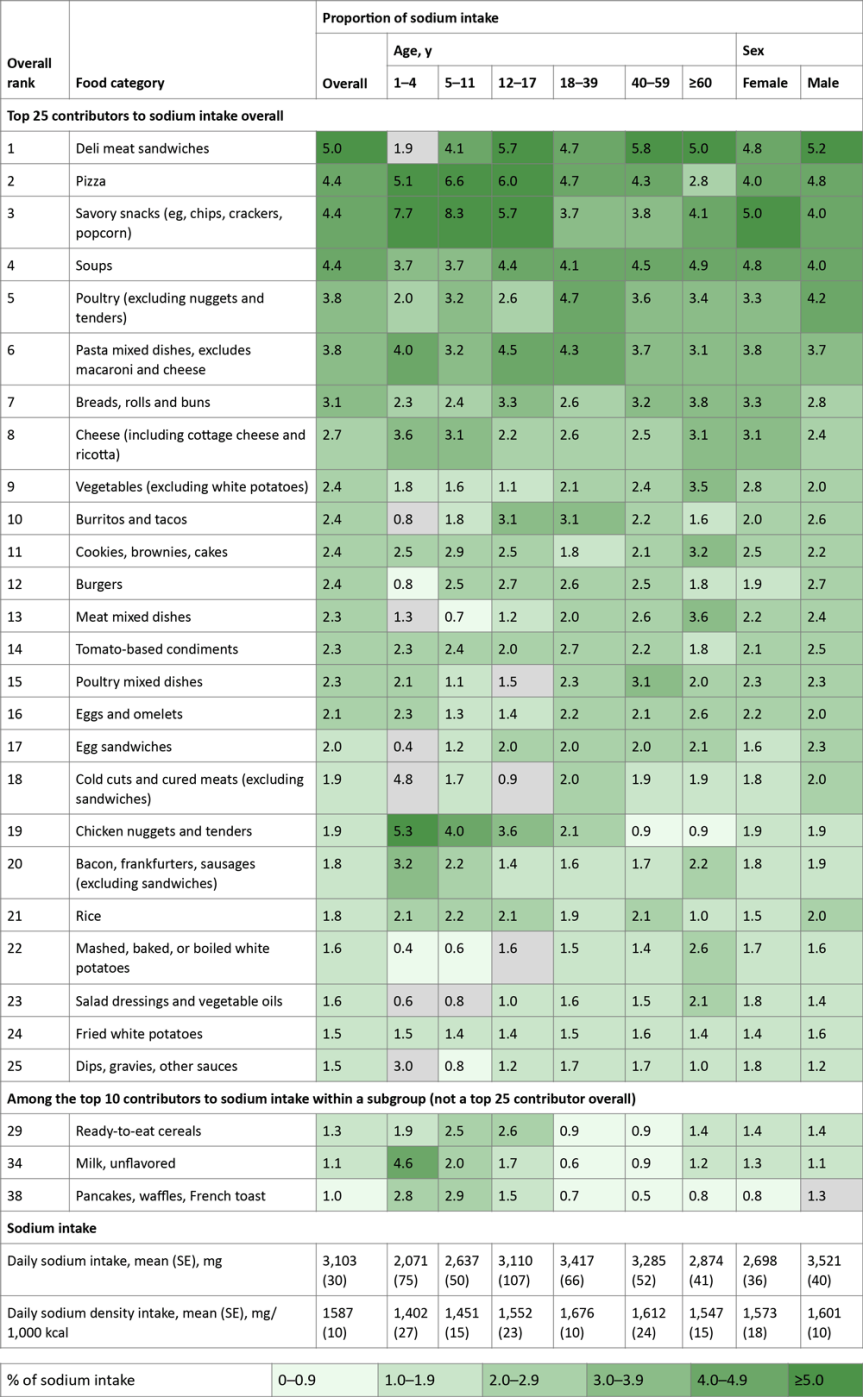
Top food category contributors to sodium intake among those aged 1 year or older, by age and sex, National Health and Nutrition Examination Survey, August 2021–August 2023. Gray shading indicates a relative SE ≥0.30; estimate is therefore statistically unreliable.

**Figure 2:**
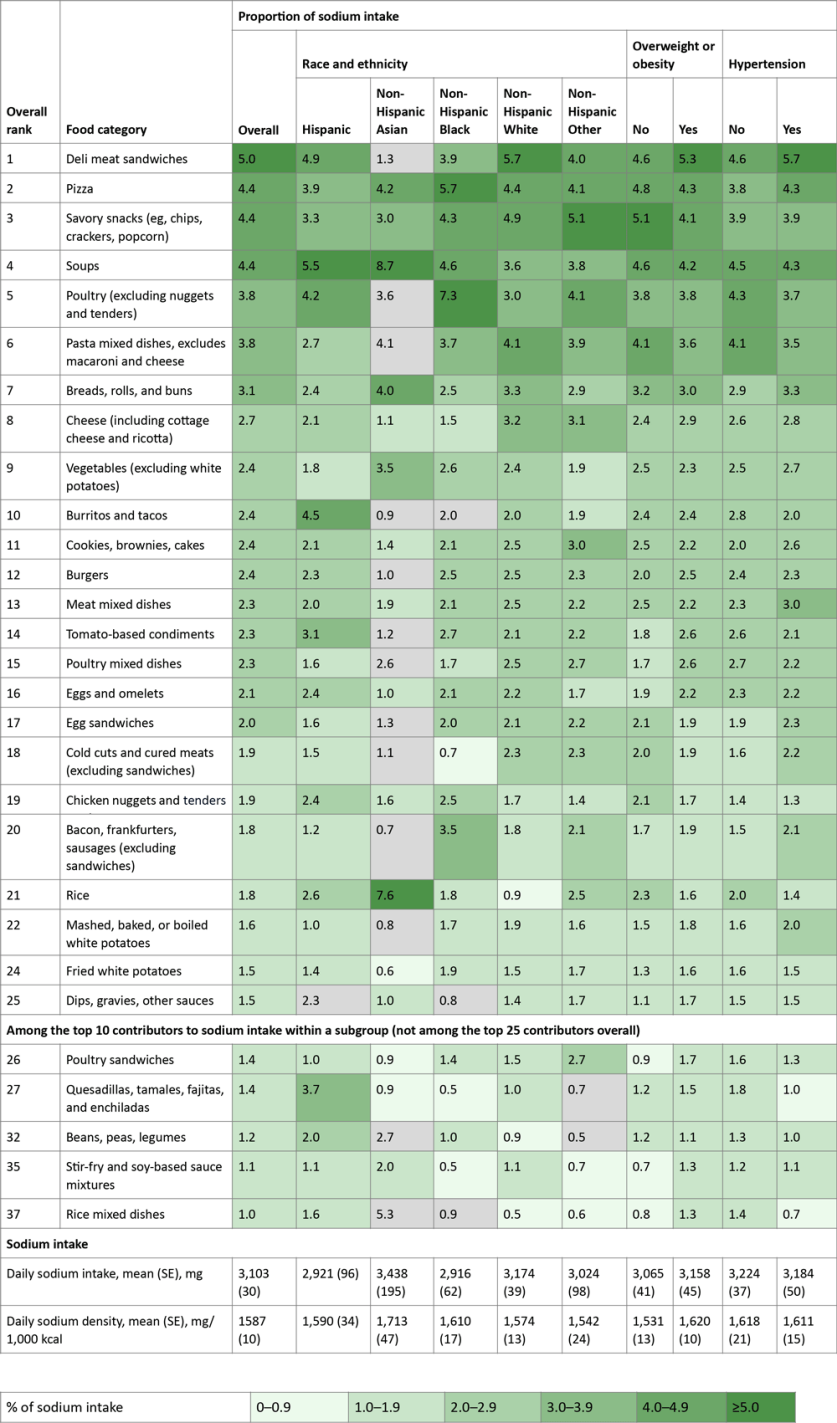
Top food category contributors to sodium intake among those aged 1 year or older, by race and ethnicity, overweight or obesity, and hypertension. National Health and Nutrition Examination Survey, August 2021–August 2023. Gray shading indicates a relative SE ≥0.30; estimate is therefore statistically unreliable.

Overall, mean (SE) daily sodium intake was 3,103 (30) mg and was highest for adults aged 18 to 39 years, males, and non-Hispanic Asian adults (Figure 1 and Figure 2).

**Table:** 

Overall rank	Food category	Proportion of sodium intake								
		Overall	Age, y						Sex	
			1–4	5–11	12–17	18–39	40–59	≥60	Female	Male
**Top 25 contributors to sodium intake overall**										
1	Deli meat sandwiches	5.0	1.9	4.1	5.7	4.7	5.8	5.0	4.8	5.2
2	Pizza	4.4	5.1	6.6	6.0	4.7	4.3	2.8	4.0	4.8
3	Savory snacks (eg, chips, crackers, popcorn)	4.4	7.7	8.3	5.7	3.7	3.8	4.1	5.0	4.0
4	Soups	4.4	3.7	3.7	4.4	4.1	4.5	4.9	4.8	4.0
5	Poultry (excluding nuggets and tenders)	3.8	2.0	3.2	2.6	4.7	3.6	3.4	3.3	4.2
6	Pasta mixed dishes, excludes macaroni and cheese	3.8	4.0	3.2	4.5	4.3	3.7	3.1	3.8	3.7
7	Breads, rolls, and buns	3.1	2.3	2.4	3.3	2.6	3.2	3.8	3.3	2.8
8	Cheese (including cottage cheese and ricotta)	2.7	3.6	3.1	2.2	2.6	2.5	3.1	3.1	2.4
9	Vegetables (excluding white potatoes)	2.4	1.8	1.6	1.1	2.1	2.4	3.5	2.8	2.0
10	Burritos and tacos	2.4	0.8	1.8	3.1	3.1	2.2	1.6	2.0	2.6
11	Cookies, brownies, cakes	2.4	2.5	2.9	2.5	1.8	2.1	3.2	2.5	2.2
12	Burgers	2.4	0.8	2.5	2.7	2.6	2.5	1.8	1.9	2.7
13	Meat mixed dishes	2.3	1.3	0.7	1.2	2.0	2.6	3.6	2.2	2.4
14	Tomato-based condiments	2.3	2.3	2.4	2.0	2.7	2.2	1.8	2.1	2.5
15	Poultry mixed dishes	2.3	2.1	1.1	1.5	2.3	3.1	2.0	2.3	2.3
16	Eggs and omelets	2.1	2.3	1.3	1.4	2.2	2.1	2.6	2.2	2.0
17	Egg sandwiches	2.0	0.4	1.2	2.0	2.0	2.0	2.1	1.6	2.3
18	Cold cuts and cured meats (excluding sandwiches)	1.9	4.8	1.7	0.9	2.0	1.9	1.9	1.8	2.0
19	Chicken nuggets and tenders	1.9	5.3	4.0	3.6	2.1	0.9	0.9	1.9	1.9
20	Bacon, frankfurters, sausages (excluding sandwiches)	1.8	3.2	2.2	1.4	1.6	1.7	2.2	1.8	1.9
21	Rice	1.8	2.1	2.2	2.1	1.9	2.1	1.0	1.5	2.0
22	Mashed, baked, or boiled white potatoes	1.6	0.4	0.6	1.6	1.5	1.4	2.6	1.7	1.6
23	Salad dressings and vegetable oils	1.6	0.6	0.8	1.0	1.6	1.5	2.1	1.8	1.4
24	Fried white potatoes	1.5	1.5	1.4	1.4	1.5	1.6	1.4	1.4	1.6
25	Dips, gravies, other sauces	1.5	3.0	0.8	1.2	1.7	1.7	1.0	1.8	1.2
**Among the top 10 contributors to sodium intake within a subgroup (not a top 25 contributor overall)**										
29	Ready-to-eat cereals	1.3	1.9	2.5	2.6	0.9	0.9	1.4	1.4	1.4
34	Milk, unflavored	1.1	4.6	2.0	1.7	0.6	0.9	1.2	1.3	1.1
38	Pancakes, waffles, French toast	1.0	2.8	2.9	1.5	0.7	0.5	0.8	0.8	1.3
**Sodium intake**										
Daily sodium intake, mean (SE), mg		3,103 (30)	2,071 (75)	2,637 (50)	3,110 (107)	3,417 (66)	3,285 (52)	2,874 (41)	2,698 (36)	3,521 (40)
Daily sodium density intake, mean (SE), mg/1,000 kcal		1587 (10)	1,402 (27)	1,451 (15)	1,552 (23)	1,676 (10)	1,612 (24)	1,547 (15)	1,573 (18)	1,601 (10)

**Table:** 

Overall rank	Food category	Proportion of sodium intake									
		Overall	Race and ethnicity					Overweight or obesity		Hypertension	
			Hispanic	Non-Hispanic Asian	Non-Hispanic Black	Non-Hispanic White	Non-Hispanic Other	No	Yes	No	Yes
1	Deli meat sandwiches	5.0	4.9	1.3	3.9	5.7	4.0	4.6	5.3	4.6	5.7
2	Pizza	4.4	3.9	4.2	5.7	4.4	4.1	4.8	4.3	3.8	4.3
3	Savory snacks (eg, chips, crackers, popcorn)	4.4and	3.3	3.0	4.3	4.9	5.1	5.1	4.1	3.9	3.9
4	Soups	4.4	5.5	8.7	4.6	3.6	3.8	4.6	4.2	4.5	4.3
5	Poultry (excluding nuggets and tenders)	3.8	4.2	3.6	7.3	3.0	4.1	3.8	3.8	4.3	3.7
6	Pasta mixed dishes, excludes macaroni and cheese	3.8	2.7	4.1	3.7	4.1	3.9	4.1	3.6	4.1	3.5
7	Breads, rolls, and buns	3.1	2.4	4.0	2.5	3.3	2.9	3.2	3.0	2.9	3.3
8	Cheese (including cottage cheese and ricotta)	2.7	2.1	1.1	1.5	3.2	3.1	2.4	2.9	2.6	2.8
9	Vegetables (excluding white potatoes)	2.4	1.8	3.5	2.6	2.4	1.9	2.5	2.3	2.5	2.7
10	Burritos and tacos	2.4	4.5	0.9	2.0	2.0	1.9	2.4	2.4	2.8	2.0
11	Cookies, brownies, cakes	2.4	2.1	1.4	2.1	2.5	3.0	2.5	2.2	2.0	2.6
12	Burgers	2.4	2.3	1.0	2.5	2.5	2.3	2.0	2.5	2.4	2.3
13	Meat mixed dishes	2.3	2.0	1.9	2.1	2.5	2.2	2.5	2.2	2.3	3.0
14	Tomato-based condiment	2.3	3.1	1.2	2.7	2.1	2.2	1.8	2.6	2.6	2.1
15	Poultry mixed dishes	2.3	1.6	2.6	1.7	2.5	2.7	1.7	2.6	2.7	2.2
16	Eggs and omelets	2.1	2.4	1.0	2.1	2.2	1.7	1.9	2.2	2.3	2.2
17	Egg sandwiches	2.0	1.6	1.3	2.0	2.1	2.2	2.1	1.9	1.9	2.3
18	Cold cuts and cured meats (excluding sandwiches)	1.9	1.5	1.1	0.7	2.3	2.3	2.0	1.9	1.6	2.2
19	Chicken nuggets and tenders	1.9	2.4	1.6	2.5	1.7	1.4	2.1	1.7	1.4	1.3
20	Bacon, frankfurters, sausages (excluding sandwiches)	1.8	1.2	0.7	3.5	1.8	2.1	1.7	1.9	1.5	2.1
21	Rice	1.8	2.6	7.6	1.8	0.9	2.5	2.3	1.6	2.0	1.4
22	Mashed, baked, or boiled white potatoes	1.6	1.0	0.8	1.7	1.9	1.6	1.5	1.8	1.6	2.0
23	Salad dressings and vegetable oils	1.6	1.0	0.8	1.5	1.9	1.1	1.6	1.6	1.8	1.7
24	Fried white potatoes	1.5	1.4	0.6	1.9	1.5	1.7	1.3	1.6	1.6	1.5
25	Dips, gravies, other sauces	1.5	2.3	1.0	0.8	1.4	1.7	1.1	1.7	1.5	1.5
**Among the top 10 contributors to sodium intake within a subgroup (not among the top 25 contributors overall)**											
26	Poultry sandwiches	1.4	1.0	0.9	1.4	1.5	2.7	0.9	1.7	1.6	1.3
27	Quesadillas, tamales, fajitas, and enchiladas	1.4	3.7	0.9	0.5	1.0	0.7	1.2	1.5	1.8	1.0
32	Beans, peas, legumes	1.2	2.0	2.7	1.0	0.9	0.5	1.2	1.1	1.3	1.0
35	Stir-fry and soy-based sauce mixtures	1.1	1.1	2.0	0.5	1.1	0.7	0.7	1.3	1.2	1.1
37	Rice mixed dishes	1.0	1.6	5.3	0.9	0.5	0.6	0.8	1.3	1.4	0.7
**Sodium intake**											
Daily sodium intake, mean (SE), mg		3,103 (30)	2,921 (96)	3,438 (195)	2,916 (62)	3,174 (39)	3,024 (98)	3,065 (41)	3,158 (45)	3,224 (37)	3,184 (50)
Daily sodium density, mean (SE), mg/1,000 kcal		1587 (10)	1,590 (34)	1,713 (47)	1,610 (17)	1,574 (13)	1,542 (24)	1,531 (13)	1,620 (10)	1,618 (21)	1,611 (15)

Foods representing at least 2.5% of total sodium intake (Figure 1) were, in descending order, deli meat sandwiches (5.0%); pizza (4.4%); savory snacks (eg, chips, crackers, popcorn, 4.4%); soups (4.4%); poultry (excluding nuggets and tenders, 3.8%); pasta mixed dishes (excluding macaroni and cheese, 3.8%); breads, rolls, and buns (3.1%); and cheese (including cottage cheese and ricotta, 2.7%) (Table).

**Table:** Characteristics of Children and Adults Aged ≥1 Year, National Health and Nutrition Examination Survey, August 2021–August 2023^a^

Characteristic	Overall (n = 6,633)		Children (aged 1–17 y) (n = 1,652)		Adults (≥18 y) (n = 4,981)	
	% (95% CI)	No.	% (95% CI)	No.	% (95% CI)	No.
**Age, y**						
1–4	4.3 (3.7–4.8)	351	20.0 (17.1–22.8)	351	—	—
5–11	9.1 (8.0–10.1)	732	42.4 (40.2–44.7)	732	—	—
12–17	8.0 (7.3–8.8)	569	37.6 (35.4–39.9)	569	—	—
18–39	29.6 (27.5–31.8)	1,301	—	—	37.7 (35.0–40.3)	1,301
40–59	25.4 (23.9–26.9)	1,328	—	—	32.3 (30.2–34.4)	1,328
≥60	23.6 (21.6–25.6)	2,352	—	—	30.0 (27.9–32.2)	2,352
**Sex**						
Female	50.8 (49.5–52.1)	3,613	48.6 (46.4–50.9)	840	51.4 (50.0–52.8)	2,773
Male	49.2 (47.9–50.5)	3,020	51.4 (49.1–53.6)	812	48.6 (47.2–50.0)	2,208
**Race and ethnicity**						
Hispanic	18.7 (12.7–24.7)	1,305	25.2 (17.9–32.5)	477	17.0 (11.2–22.8)	828
Non-Hispanic Asian	5.7 (3.4–7.9)	327	7.7 (4.6–10.8)	113	5.1 (3.0–7.2)	214
Non-Hispanic Black	11.6 (8.6–14.5)	823	12.1 (9.0–15.1)	244	11.4 (8.2–14.6)	579
Non-Hispanic White	57.9 (53.6–62.3)	3,716	46.6 (40.3–52.9)	669	61.0 (57.0–65.0)	3,047
Non-Hispanic Other^b^	6.1 (5.0–7.2)	462	8.4 (6.1–10.8)	149	5.5 (4.3–6.7)	313
**Overweight or obesity** c						
No	34.4 (31.5–37.2)	2,314	60.1 (57.5–62.7)	968	27.4 (24.2–30.5)	1,346
Yes	63.5 (60.6–66.4)	4,156	33.1 (29.4–36.8)	578	71.7 (68.6–74.9)	3,578
Missing	2.1 (1.9–2.4)	163	6.8 (4.9–8.6)	106	0.9 (0.6–1.1)	57
**Hypertension** d						
No	—	—	—	—	51.2 (48.9–53.6)	2,219
Yes	—	—	—	—	47.2 (45.0–49.5)	2,693
Missing	—	—	—	—	1.5 (1.0–2.0)	69

Abbreviation: —, does not apply.

a Reported values were estimated using the “survey” package in R version 4.5.0 (R Foundation), adjusting for the complex survey design and estimating variance using Taylor series linearization. Survey weights from day 1 dietary recall were used.

b Includes American Indian/Alaska Native, Native Hawaiian/Other Pacific Islander, and “other.”

c Overweight or obesity was based on body mass index ≥85th percentile for individuals aged 2–19 years based on age- and sex-specific thresholds provided by the Centers for Disease Control and Prevention (8). For those aged ≥20 years, overweight or obesity was based on body mass index ≥25 kg/m^2^, based on criteria from the World Health Organization (9).

d Hypertension was defined as a systolic pressure ≥130 mm Hg or diastolic pressure ≥80 mm Hg (based on criteria from the American Heart Association) (10) or self-reported use of hypertension medication. Hypertension was not evaluated for children aged ≤17 years.

Generally, the top food category contributors to sodium intake across age groups were the same, with some exceptions. For example, among children aged 1 to 11 years, savory snacks were the largest contributor to sodium intake, at approximately 8%, but among other age groups, savory snacks made up a smaller proportion of sodium intake. Among children aged 1 to 4 years, unflavored milk was the fifth-largest contributor to sodium intake (4.6%), but among other age groups, unflavored milk made up 0.6% to 2.0% of sodium intake.

The distribution of top contributors varied by race and ethnicity (Figure 2). Among Hispanic participants, the quesadillas, tamales, fajitas, and enchiladas food category represented 3.7% of sodium intake and was the sixth-largest contributor to sodium intake yet was not among the top 25 contributors overall. Among non-Hispanic Asian participants, soups and rice were the top 2 contributors, representing 16.3% of total sodium intake. Among non-Hispanic Black participants, poultry was the top contributor (7.3%), while among participants from the other racial and ethnic groups, poultry represented 3.0% to 4.2% of sodium intake.

## Discussion

In a nationally representative sample of children and adults, the top contributors to sodium intake were generally similar across age groups for adults, with some differences across age groups among children and by racial and ethnic group. Most top contributors to sodium intake were the same as those found in prior NHANES studies (3,4). Rankings of the top contributors to sodium intake differed slightly from the rankings found in prior NHANES studies, although it is unclear to what extent differences are due to actual dietary changes, random variation, and/or changes in the food categories used by NHANES.

Limitations include self-reported dietary data, which are subject to recall bias. Sodium intake might be misclassified in some instances; it might be underestimated because salt added at the table was excluded from dietary recalls or overestimated because USDA data assumes salt was added to certain foods (eg, rice). Although survey weights adjusted for nonresponse bias, this bias might have affected results, because the rate of participation in the examination was 25.7% (5). Results may not be comparable with other studies because of different methods of categorizing foods. Lastly, estimates for food categories in some groups, particularly children aged 1 to 4 years and non-Hispanic Asian participants, were not statistically reliable due to insufficient sample sizes.

Our findings might be used to tailor interventions to more effectively reduce sodium intake. Because population-level interventions can have the greatest impact on reducing sodium intake (11), knowledge of the top contributors of sodium intake might be helpful to consumers seeking lower-sodium products and might support food manufacturers’ implementation of the voluntary sodium reduction goals of the US Food and Drug Administration (12). Bread and bread-containing foods made up multiple top food categories contributing to sodium intake, and salt in bread may be reduced by approximately 40% without substantially affecting consumer acceptability (13). Moreover, because a wide variety of foods contributes to sodium intake, interventions can focus on multiple foods rather than any single food. When top contributors to sodium intake among certain demographic groups differ from those seen among the overall population, interventions can be tailored appropriately. For example, interventions for children can focus on lower-sodium alternatives for savory snacks served in settings such as early care and education facilities and schools. Information on top contributors of sodium intake might enhance schools’ capacity to meet recently updated federal guidelines on sodium limits for school meals (14). At the individual level, increased education and awareness might help consumers identify their greatest sources of sodium intake. Counseling by health care providers and consumer education efforts at reducing dietary sodium can complement other food system–level interventions (15).
